# Effect of Dentin Bonding Agent on the Prevention of Tooth Discoloration Produced by Mineral Trioxide Aggregate

**DOI:** 10.1155/2012/563203

**Published:** 2011-11-03

**Authors:** Majid Akbari, Armita Rouhani, Sadeq Samiee, Hamid Jafarzadeh

**Affiliations:** ^1^Department of Restorative Dentistry, School of Dentistry, Mashhad University of Medical Sciences, Mashhad 91735-984, Iran; ^2^Department of Endodontics, School of Dentistry, Mashhad University of Medical Sciences, Mashhad 91735-984, Iran

## Abstract

*Objective*. Determination of the effect of dentin bonding agent (DBA) on the prevention of tooth discoloration produced by mineral trioxide aggregate (MTA). *Methods*. 50 teeth were endodontically treated and after removal of 3 mm of obturating materials were divided into five groups. In white MTA (WMTA) and grey MTA (GMTA) groups, these materials were placed in root canal below the orifice. In DBA + WMTA and DBA + GMTA groups, DBAs were applied in the access cavity. Then, 3 mm of WMTA and GMTA was placed. The last 10 teeth served as control. All of teeth were restored and color measurement was recorded for each specimen at this time and 6 months later. *Results*. The mean tooth discoloration in WMTA and GMTA groups was significantly more than DBA + WMTA and DBA + GMTA groups, respectively. There was no significant difference between DBA + WMTA and DBA + GMTA groups and control group. *Conclusion*. Application of DBA before MTA may prevent tooth discoloration.

## 1. Introduction

Mineral trioxide aggregate (MTA) is a biocompatible material with high sealing ability which has been used for various purposes in endodontics, including root end filling, sealing of perforations, treatment of teeth with open apices, and as a pulp capping agent [1–3].

The color of original MTA (grey MTA or GMTA) was grey and it had a potential of tooth discoloration. Because of this disadvantage, white MTA (WMTA) introduced in order to this problem be solved [4]. Asgary et al. [5] showed that the major differences in chemical component between WMTA and GMTA are concentrations of Al_2_O_3_, MgO, and FeO. Parirokh et al. [6] found that there were no significant differences in pulp responses to both types of MTA when used as a pulp capping agent in dog's teeth. Holland et al. [7] also found that the mechanisms of action of WMTA and GMTA are similar. However, Matt et al. [8] found that GMTA has significantly less leakage than WMTA when used as an apical barrier.

Although WMTA was developed to solve the problem of tooth discoloration produced by GMTA, several studies have reported tooth discoloration after using both kinds of MTA [9–15]. This effect limits MTA application in treatment of perforations, pulp capping, pulpotomy, and as an apical barrier in aesthetically sensitive areas.

Bonding to dentin is one of the most significant advances in the past fifty years. The success of this kind of bonding has depended more on creative chemistry than etching with some materials such as phosphoric acid. Many generations of dentin bonding agents (DBAs) have been produced. With new advances in new material's technology, bonding to dentin has been reported to be favorable [16].

The purpose of this study was to evaluate the effect of the application of DBA before usage of MTA to prevent tooth discoloration.

## 2. Materials and Methods

Fifty freshly extracted single-rooted human maxillary central and lateral incisors were used in this study. The teeth were clinically and radiographically examined to be free of caries, cracks, restoration, and calcification. External surfaces of the teeth were cleaned with curettes and stored in a physiologic saline solution until usage. 

An access cavity was prepared with a number 4 round bur. The pulp tissue was removed by using barbed broaches (Dentsply Maillefer, Tulsa, Okla, USA). Working length was determined visually with stainless steel hand files (Dentsply Maillefer, Tulsa, Okla, USA) trough the canal until the tip was seen at apical foramen and working length calculated by subtracting 1 mm from this length. Each canal was prepared by using K-files (Dentsply Maillefer, Tulsa, Okla, USA) and gates-glidden drills (Dentsply Maillefer, Tulsa, Okla, USA) in a step back manner. The apical area was prepared up to K-File number 40. Irrigation was carried out by using 2.5% NaOCl. After drying root canal with paper points, master gutta-percha was placed at canal and confirmed radiographically, and teeth were obturated using gutta-percha (Arya Dent, Tehran, Iran) and AH-Plus sealer (Dentsply, Konstanz, Germany) by lateral condensation technique. Then the extruded cones were cut off 3 mm below the orifice and compacted vertically. 

Teeth were randomly divided into five groups. In groups WMTA and GMTA, 3 mm of white and grey MTA (MTA Angelus, Londrina, PR, Brazil) plug was placed in root canal below the orifice, respectively. In groups DBA + WMTA and DBA + GMTA, two layers of DBA (Clearfil SE Bond, Kurary, Okayama, Japan) was applied in access cavity and light cured for 40 seconds and then 3 mm of WMTA and GMTA was placed in root canal below the orifice, respectively. 

After complete cleaning of the access cavity, a moistened cotton pellet was placed in access cavity and coronal access was sealed with coltozol (Coltene, Altstatten, Switzerland) for 24 hours. These teeth were kept in wet gauze. After that, temporary filling was removed and the teeth were restored with resin composite (z100, 3M, USA). The last 10 teeth served as control, with no DBA and MTA and restored with resin composite. 

At the baseline, color measurement of all teeth was recorded with a colorimeter (Minolta CR-300; Minolta, Osaka, Japan). Measurements were repeated 3 times for each specimen, and the mean values of data were calculated. 

The teeth were kept in artificial saliva for 6 months, whereas artificial saliva was replenished each two weeks. At this point, color readings were made using the colorimeter in the manner described for baseline readings. The calculation of the color variation ΔE* between the 2 color measurements is as follows:

(1) $$\Delta E^{*}={\left[{\left(\Delta L^{*}\right)}^{2}+{\left(\Delta a^{*}\right)}^{2}+{\left(\Delta b^{*}\right)}^{2}\right]}^{1/2}.$$

*L* refers to the lightness coordinate with value ranging from zero (black) to 100 (white). The values *a* and *b* are chromaticity coordinates in the red-green axis and the yellow-blue axis, respectively [17]. 

Preliminary analysis with Kolmogorof-Smirnov test was used to confirm the normal distribution of the data. The results were analyzed by *t*-test, with the significance level defined as *α* = 0.05.

## 3. Results

**Figure 1:**
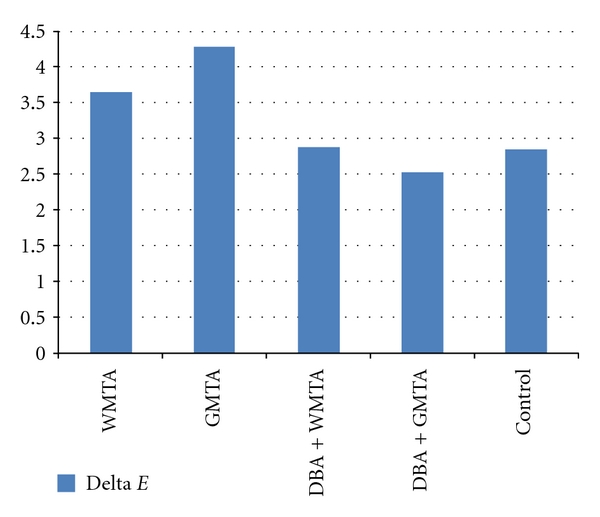
Mean tooth discoloration in 5 groups after 6 months.

All teeth showed discoloration after 6 months. The mean discoloration after 6 months is shown in Figure 1. The mean tooth discoloration in WMTA group was significantly more than DBA + WMTA and control groups. There was no significant difference between DBA + WMTA group and control group in mean discoloration.

The teeth in GMTA group also showed significantly more discoloration than DBA + GMTA and control groups. No significant difference was observed between control and DBA + GMTA groups. 

There was no significant difference between GMTA group and WMTA group in mean tooth discoloration, as well as for DBA + GMTA and DBA + WMTA groups.

## 4. Discussion

MTA has been recognized as a bioactive material [18] that is hard tissue conductive [19], hard tissue inductive, and biocompatible [20], so the applications of this material have been rapidly expanding in dentistry. Despite such good characteristic, MTA has some drawbacks including discoloration potential, difficult handling properties, long setting time, high cost, and absence of a known solvent [21]. 

Our study revealed that when GMTA was placed below the root canal orifice, the crown of teeth, especially in the cervical area, showed significant tooth discoloration after 6 months. This result was coinciding with other studies that showed tooth discoloration after using GMTA [9, 10, 13]. 

Due to the potential discoloration of teeth treated with GMTA, the manufacturer introduced a new formula of MTA with an off-white color. However, this study showed that the application of WMTA may also cause tooth discoloration. This can be attributed to the fact that although the concentration of carborundum (Al_2_O_3_), periclase (MgO), and FeO has been lowered in WMTA compared to GMTA [5], these metal oxides are still present in WMTA and can cause tooth discoloration. Jacobovitz and De Lima [11] used WMTA for the treatment of inflammatory internal root resorption. They observed grey discoloration of teeth after 20 months. Boutsioukis et al. [12] which examined the efficiency of two techniques for removal of WMTA from root canals also reported dark discoloration of most MTA fillings.

Application of two layers of DBA before using MTA may prevent tooth discoloration produced by both WMTA and GMTA. This can be related to the sealing ability of DBA that seals dentinal tubules in access cavity and below the orifice before MTA application. This process makes a surface that prevents any contamination and remaining MTA powder in the access cavity during insertion. Remaining powder has a potential to degrade and make colorant material such as FeO. 

According to the results of this study, it can be recommended to seal dentinal tubules by DBA before using both MTAs to prevent further tooth discoloration. Since DBA may interfere with proper sealing ability of MTA or have a possible interference with the release of calcium via dentin tubules, it is advisable to conduct other studies to evaluate the effect of DBA on these properties of MTA.
